# Clinical and radiographic assessment of peripheral joints in controlled acromegaly

**DOI:** 10.1007/s11102-022-01233-z

**Published:** 2022-06-20

**Authors:** Iris C. M. Pelsma, Herman M. Kroon, Victoria R. van Trigt, Alberto M. Pereira, Margreet Kloppenburg, Nienke R. Biermasz, Kim M. J. A. Claessen

**Affiliations:** 1 Department of Medicine, Division of Endocrinology and Metabolism, and Center for Endocrine Tumors Leiden, Leiden University Medical Center, Leiden, The Netherlands; 2 Department of Radiology, Leiden University Medical Center, Leiden, The Netherlands; 3 Department of Endocrinology and Metabolism, Amsterdam University Medical Center, Amsterdam, The Netherlands; 4 Department of Rheumatology, Leiden University Medical Center, Leiden, The Netherlands; 5 Department of Epidemiology, Leiden University Medical Center, Leiden, The Netherlands

**Keywords:** Acromegaly, Growth hormone, Insulin-like growth factor-1, Arthropathy, Osteoarthritis, Shoulder

## Abstract

**Purpose:**

Acromegalic arthropathy is a well-known phenomenon, occurring in most patients regardless of disease status. To date, solely hips, knees, hands, and spinal joints have been radiographically assessed. Therefore, this study aimed to assess the prevalence of joint symptoms and radiographic osteoarthritis (OA) of new, and established peripheral joint sites in well-controlled acromegaly.

**Methods:**

Fifty-one acromegaly patients (56% female, mean age 64 ± 12 years) in long-term remission for 18.3 years (median, IQR 7.2–25.4) were included. Nineteen patients currently received pharmacological treatment. Self-reported joint complaints were assessed using standardized interviews. Self-reported disability of the upper and lower limbs, and health-related quality of life (HR-QoL) were evaluated using validated questionnaires. Radiographic OA [defined as Kellgren & Lawrence (KL) ≥ 2] was scored using (modified) KL methods.

**Results:**

Radiographic signs of OA were present in 46 patients (90.2%) with ≥ 2 joints affected in virtually all of these patients (N = 44; 95.7%). Radiographic MTP1 OA was as prevalent as radiographic knee OA (N = 26, 51.0%), and radiographic glenohumeral OA was similarly prevalent as hip OA [N = 21 (41.2%) *vs.* N = 24 (47.1%)]. Risk factors for radiographic glenohumeral OA were higher pre-treatment IGF-1 levels [OR 1.06 (1.01–1.12), *P* = 0.021], and current pharmacological treatment [OR 5.01 (1.03–24.54), *P* = 0.047], whereas no risk factors for MTP1 joint OA could be identified.

**Conclusion:**

Similar to previously-assessed peripheral joints, clinical and radiographic arthropathy of the shoulder and feet were prevalent in controlled acromegaly. Further studies on adequate management strategies of acromegalic arthropathy are needed.

**Supplementary Information:**

The online version contains supplementary material available at 10.1007/s11102-022-01233-z.

## Introduction

Acromegaly is characterized by growth hormone (GH) and Insulin-like growth factor-1 (IGF-1) excess, resulting in a plethora of clinical complaints [1–5]. Multimodality treatment strategies—surgical adenoma resection, radiotherapy, and pharmacological treatment— result in disease control in most patients, with concomitant improvement of symptoms, and comorbid conditions. Nonetheless, patients may suffer from (partially) irreversible, persisting, or delayed complaints [5, 6].

One of the most invalidating skeletal acromegalic complications is arthropathy, affecting both peripheral and axial joints [3, 7, 8]. Compared to the general population, the prevalence of acromegalic arthropathy is 2–9 times higher—depending on the joint site—despite achievement of biochemical remission [3]. Acromegaly patients frequently report joint pain, stiffness, and functional limitations, with arthropathy contributing to impaired health-related quality of life (HR-QoL) [6]. Additionally, upon radiographic evaluation, acromegalic arthropathy displays a unique phenotype with severe osteophytosis (OP), and distinctive joint space widening (JSW), differing significantly from primary osteoarthritis (OA) that is characterized by joint space narrowing (JSN) due to cartilage loss [9–12]. Progression of arthropathy, clinically or radiographically, has been reported for a significant proportion of patients, independent of disease remission [13–15]. Patients with higher age, higher baseline IGF-1 levels, treatment with somatostatin (SMS) analogs required for disease control [13], and patients with increased severity of OA at baseline [15] were at increased risk for radiological OA progression.

Previous studies on acromegalic arthropathy investigated hips, knees, hands, and spine joints [2, 3, 6, 7, 13–15], whereas literature on other joints, e.g. shoulders and feet, is scarce. Shoulder arthropathy can be invalidating in nature, with individuals with shoulder complaints reporting significant impairments in HR-QoL [16–19]. Previously, self-reported shoulder complaints have been evaluated in acromegaly [8, 20, 21], whereas radiographic shoulder OA has not been assessed in patients with acromegaly. Moreover, although enlargement of the feet is frequently reported as an early symptom of acromegaly, and heel tendinopathy is observed in up to 50% of patients [12, 22], arthropathy of the feet has not been systematically evaluated [23]. Moreover, since OA in the first metatarsophalangeal (MTP1) joint is the most common presentation of foot OA in the general population, which is associated with significant locomotor disability [24], and decreased health-related QoL [25], this joint is very relevant to investigate in patients with acromegaly.

Therefore, in the present study, the prevalence, and potential risk factors of acromegalic arthropathy of the shoulder and feet in a cohort of biochemically controlled acromegaly patients were assessed. Moreover, these newly assessed joints were compared to previously-assessed peripheral joints (viz*.* hands, hips, knees). Furthermore, the effects of radiographic OA on HR-QoL in the present population were investigated. Ultimately, we propose a clinical-practice-based algorithm for the diagnosis and management of acromegalic arthropathy.

## Methods

### Study design and patient selection

#### Study protocol

This cross-sectional study was approved by the Medical Ethics Committee of the Leiden University Medical Center (LUMC), and all patients gave written informed consent prior to participation. As reported previously [26], all patients completed a clinical standardized interview, validated questionnaires, and the assessment of radiographic OA in peripheral joints (vide infra). Moreover, current serum GH and IGF-1 levels were assessed in fasting blood samples.

#### Patients

Fifty-one patients with well-controlled acromegaly were included, as described prior, for the present cross-sectional study [26]. Briefly, 31 patients, included in a prospective, longitudinal follow-up study assessing skeletal complications in patients with long-term controlled acromegaly [15, 27], and 20 consecutive, newly-included patients were combined in the present study. For the patients in the longitudinal study, the present study reported on data collected during the 10-year follow-up visit [15, 27], whereas for the newly included patients, the present study visit was the first and only study visit. All included patients were in remission at the time of inclusion, visiting the outpatient clinic of the Center for Endocrine Tumors Leiden (CETL) of the LUMC. Previously, the patient selection [26], as well as details on diagnosis, treatment, and clinical follow-up, has been described prior [13–15, 26–28]. Briefly, patients were predominantly treated by transsphenoidal surgery, with additional multimodality treatment options consisting of radiotherapy, pharmacological treatment with SMS analogues or pegvisomant (PegV), or combination therapy when necessary [6, 29, 30].

### Study parameters

#### Acromegaly disease parameters

Acromegalic disease activity and pituitary axes function were assessed annually in all patients, or more frequently when applicable, as described previously [2, 3, 7, 13, 14, 27]. Since most patients were in long-term remission, disease remission definitions have varied according to contemporaneous guidelines and reference ranges of assays. In summary, when IGF-1 levels [using age-adjusted SD scores (SDS)], and glucose-suppressed GH levels were normal, acromegaly was considered in remission, independent of treatment modality. Duration of active disease and disease remission were calculated using the estimated date of disease onset, date of serum IGF-1 levels normalization, and date of the study visit [28, 31].

#### Assessment of pituitary and gonadal function

Hypopituitarism—hormonal deficiency of ≥ 1 pituitary-end organ axis requiring supplementation—was defined according to previously published definitions [32, 33], and patients were adequately treated with replacement therapy when necessary. In detail, hormonal deficiencies were defined as: (1) thyroid stimulating hormone (TSH), free thyroxine 4 (fT4) levels below the reference range (< 10 pmol/L); (2) adrenocorticotropic hormone (ACTH), corticotropin-releasing hormone (CRH) stimulation or insulin tolerance test with insufficient increase in cortisol levels (< 0.55 μmol/L); (4) hypogonadism: male patients with testosterone concentration < 8.0 nmol/L for > 1 year, and female patients with natural menopause, or prolonged untreated amenorrhea with serum estradiol concentrations < 70 nmol/L, and (5) GH: a peak GH response of < 3 ug/L during insulin tolerance testing (ITT) with a glucose of < 2.2 mmol/L, or GHRH/arginine test (using BMI-adjusted cut-off values) when ITT is contraindicated. Patients with adequately treated hypogonadism, female patients with a normal spontaneous menstrual cycles, or female patients on estrogen hormone replacement therapy/oral contraceptives were considered eugonadal. GH deficiency was assessed only in case of clinical suspicion, and treated with an individualized dose of recombinant GH according to current guidelines [34–36].

#### Biochemical assays

Throughout the study duration, different GH and IGF-1 assays were used for biochemical assessment. Detailed descriptions of the varying GH, and IGF-1 assays have been published previously [2, 3, 7, 13–15, 27, 28]. Since 2017, serum GH levels (ug/L) were assessed using a nationally harmonized immunoassay on the IDS-iSYS analyzer with a harmonization factor of 1.02 [37]. Serum IGF-1 concentrations were measured using the IDS-iSYS immunoanalyzer, since 2014. Serum IGF-1 levels were reported as absolute levels (nmol/L), and standard deviation scores (SDS), using λ-μ-σ smoothed age- and sex-related reference curves [38, 39].

#### Standardized interview and validated questionnaires

Patients completed an interview with standardized questions on demographic data, medical history, and signs and symptoms of OA. Specifically, patients were asked for current pain or stiffness of the assessed joints (viz*.* shoulder, hands, hips, knees, and feet, respectively).

Validated questionnaires on self-reported joint symptoms were filled out. The *Disabilities of the Arm, Shoulder and Hand* (DASH, Dutch version) was used to assess physical function, and symptoms of the upper limb during the previous 7 days, using 30 questions with a 5-point Likert scale [40–43]. Total scores ranged from 0 to 100, with higher scores signifying greater disability. To evaluate of hand symptoms in the previous 48 h, the *Australian/Canadian Osteoarthritis Index* (AUSCAN) was used [44], of which all items are rated on a 5-point Likert scale ranging from 0 (none) to 4 (extreme). Total (sub)scores ranged from 0 to 60 (pain 0–20; stiffness 0–4; function 0–36). Finally, the *Western Ontario and McMaster Universities Osteoarthritis Index* (WOMAC) assessing pain, stiffness and disability of the lower limb during the previous 48 h was used [45]. Total scores ranged from 0 to 300, with subscores (pain, stiffness, and function) ranging from 0 to 100 using a 100 mm visual analog scale (VAS), and higher scores indicating more complaints.

Moreover, the validated *Short Form-36* (SF-36) was filled out to assess patients’ general HR-QoL during the previous 30 days. Thirty-six questions accompanied by standardized response choices measured eight HR-QoL domains. Total scores ranged from 0 to 100 for all domains, with higher scores reflecting higher HR-QoL [46, 47]. Physical health component score (PCS), and mental health component score (MCS) were calculated using Z-scores obtained by comparison of the eight domain scores in comparison with a general American population (sample of 2393 individuals) [48].

#### Radiographic protocol

Conventional radiographs of the hands, knees, and hips were obtained according to standardized protocols with a fixed film-focus distance and fixed joint position by a single experienced radiology technician, as reported previously [15]. For assessment of glenohumeral OA, antero-posterior (AP) in exorotation, and axial radiographs (AP) were obtained. To enable scoring of forefoot joints, dorsoplantar (DP), and DP at 45 degrees dorsal inclination radiographs of the forefoot were obtained. All radiographs were blinded for patient characteristics.

#### Assessment of radiographic OA

As reported previously, hands, hips, and knees were scored in consensus using a modified, semi-quantitative Kellgren and Lawrence (KL) scoring system by a team of two experienced assessors (K.C. and H.K.), of whom one is a musculoskeletal radiologist (H.K.) [15, 49]. The KL scoring system is based on evaluating the presence of OP, JSN, sclerosis, and degenerative cysts in a specific joint, resulting in a composite score ranging from 0 to 4 on a 5-point Likert scale [49]. Additionally, specific structural joint alterations or deformities observed during scoring, which were not reflected in the KL scoring system, were noted. Scored joints in the hands were the distal interphalangeal (DIP), proximal interphalangeal (PIP), metacarpophalangeal (MCP), first interphalangeal (IP1), and first carpometacarpal (CMC1) joints.

**Fig. 1:**
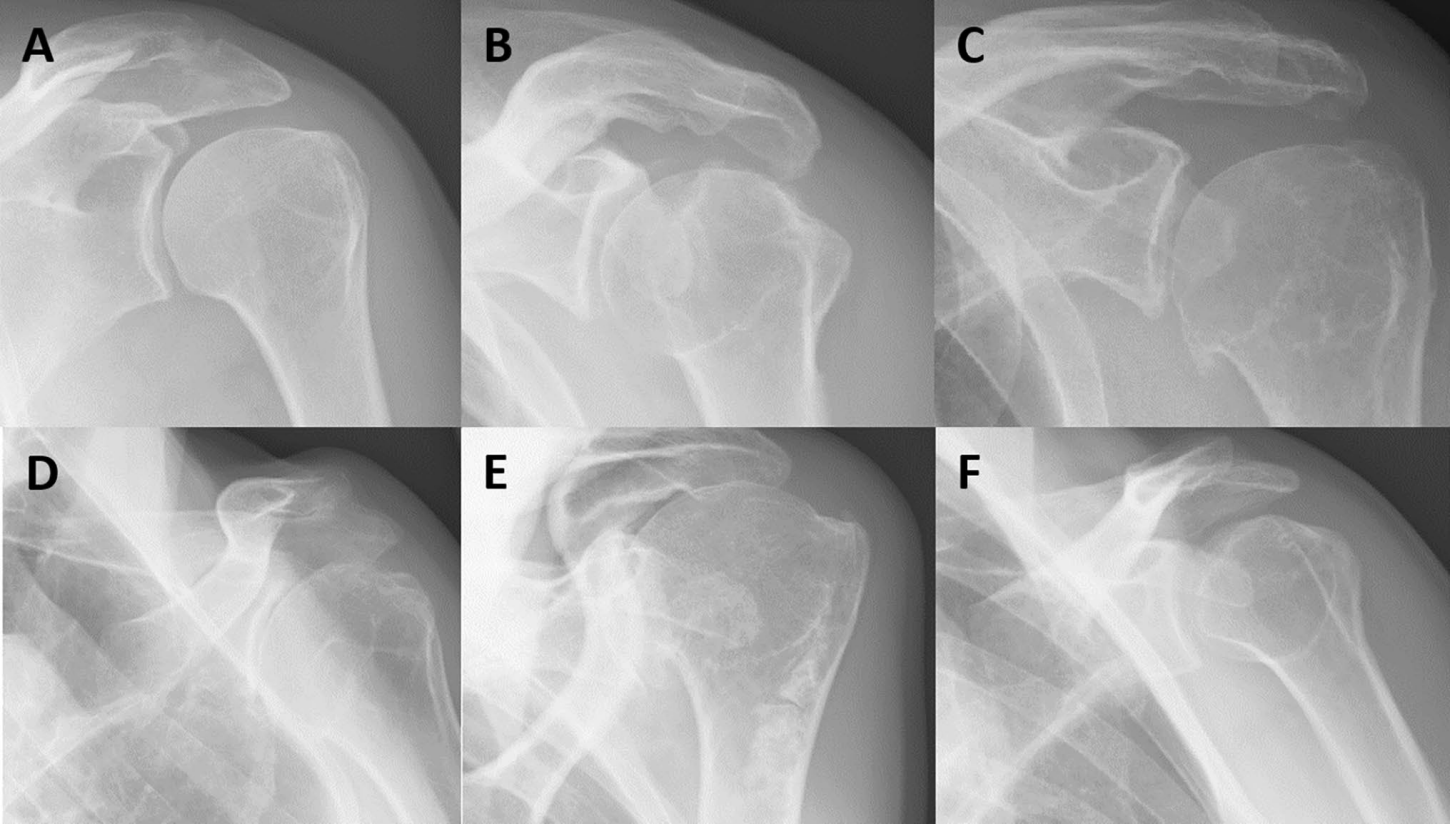
Radiographic glenohumeral OA in patients with controlled acromegaly according to a modified Kellgren and Lawrence scoring method. Examples of the different scores of the modified Kellgren and Lawrence (KL) scoring system of the glenohumeral joint in our acromegaly patients, based on previously described definitions [49–53]. **A** KL score 0, **B** KL score 1, **C** KL score 2, **D** KL score 3, and **E** KL score 4. **F** Significant joint space widening (JSW), characteristic for acromegalic arthropathy

**Fig. 2:**
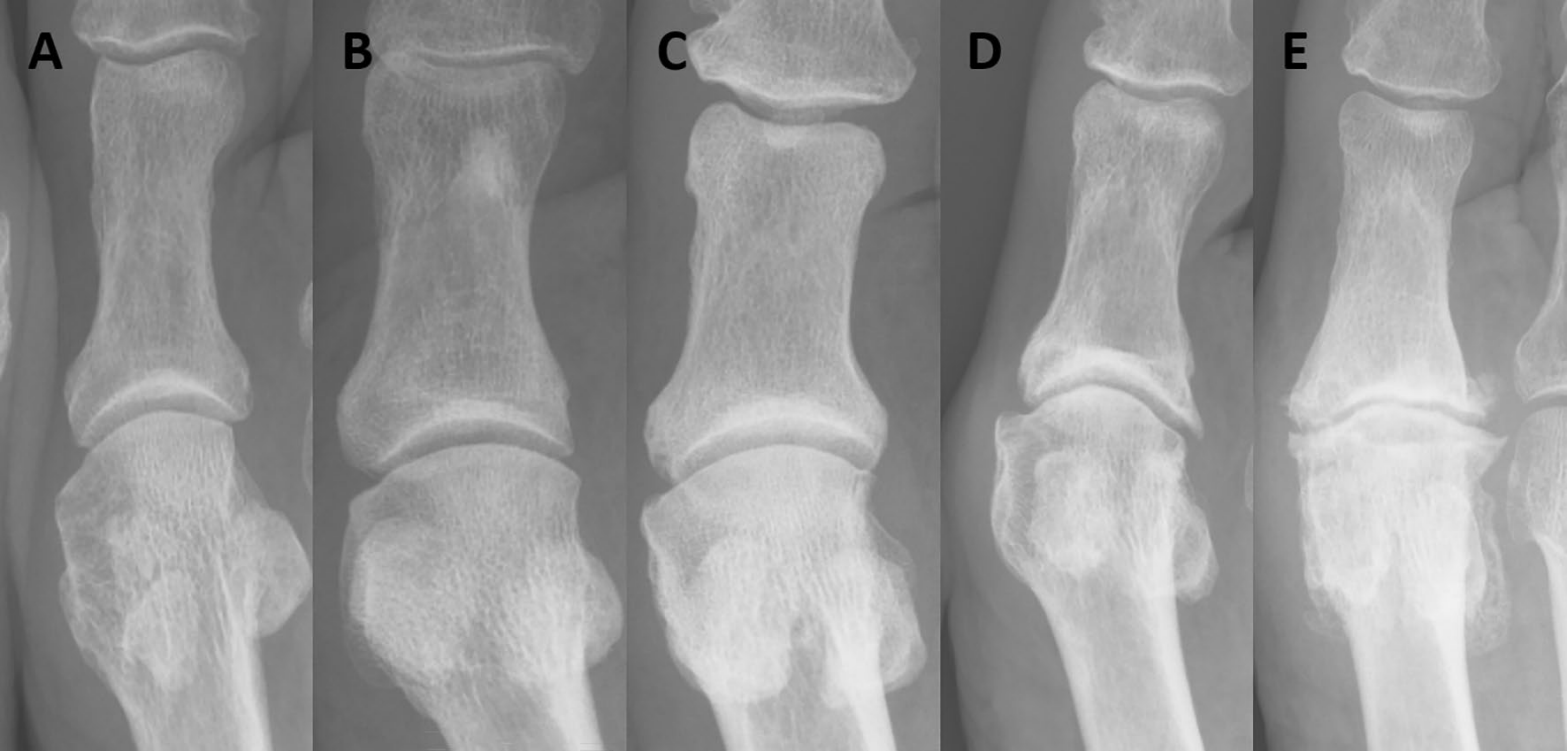
Radiographic OA of the MTP1 joint in patients with controlled acromegaly according to a modified Kellgren and Lawrence scoring method. Examples of the different scores of the modified Kellgren and Lawrence (KL) scoring system of the MTP1 joint in our acromegaly patients, based on the KL atlas of the hands, as described in several previous reports [49, 54, 55]. **A** KL score 0, **B** KL score 1, **C** KL score 2, **D** KL score 3, and **E** KL score 4. *MTP* metatarsophalangeal

Since, to date, no official KL atlas for the shoulder and forefoot joints exists, glenohumeral joints, and MTP1-5 and IP1 joints were semi-quantitively scored according to a modified KL scoring system based on the KL atlas of the knee and hands (especially MCP joints), respectively, similar to several previous studies [49–55]. KL scores were defined as follows: 0, normal bone contour, no JSN, no OP, no sclerosis; 1, doubtful JSN, possible OP; 2, definite OP and possible JSN; 3, multiple moderate OP, definite JSN, some sclerosis, and possible bone contour deformity; 4, severe OP, marked JSN, severe sclerosis, and definite bone contour deformity. Examples of the different KL scores of the glenohumeral, and MTP1 joint, as well as—characteristic for acromegaly—JSW, in our patients are shown in Figs. 1 and 2, respectively. Patients who underwent joint replacement surgery received a KL score of 4 as reflection of severe radiographic OA.

Reproducibility of the KL scores was assessed by the intra-class correlation coefficient (ICC; 95% confidence interval), based on the repeat examination in consensus of 3–6 radiographs (depending on the joint site), that were selected at random. Reproducibility scores were high: hips 1.00, knees 1.00, hands 0.97 (0.95–0.98) [CMC1: 0.80 (0.33–0.96), MCPs: 0.99 (0.98–1.00), PIPs: 0.97 (0.94–0.99), DIPs: 1.00], shoulder 0.99 (0.96–0.99), and forefoot 0.97 (0.95–0.98).

#### Diagnosis of radiographic OA

Radiographic OA was defined in multiple ways: (1) radiographic OA of individual joints with a KL score of ≥ 2; (2) radiographic OA on patient level (based on joints with KL ≥ 2), according to the presence of no, unilateral or bilateral radiographic OA, and (3) severity of radiographic OA based on total KL scores by adding left and right joints. Total KL scores range: glenohumeral 0–8; hands 0–120; hips 0–8; knees 0–8; forefoot: 0–48; peripheral: 0–192.

### Statistical analysis

SPSS for Windows version 25.0 (SPSS Inc., Chicago, IL, USA) was used for all analyses. Data were reported as number of patients [N; percentage (%)], mean ± standard deviation (SD), or median [interquartile range (IQR)]. Via two-way mixed models for single measurements, ICCs were calculated. Correlation analyses were performed using Pearson’s correlation analysis. For risk factor analyses, χ^2^ tests, Fisher’s exact tests, or binary logistic/linear regression analyses were performed. In primary OA, age, female sex, and BMI were risk factors for both radiographic OA of the glenohumeral joint [56–59], and MTP1 joint [60, 61], and were therefore assessed in the regression analyses. *P*-values < 0.05 were considered significant.

## Results

### Patient characteristics

Fifty-one patients (mean age 64 ± 12 years, 57% female) with controlled acromegaly, who were in remission for a median of 18.3 years (IQR 7.2–25.4; range 2 months to 37.5 years), were included in this study. Clinical characteristics are summarized in Table 1.

**Table 1:** Clinical characteristics of the patient population

Characteristic	All patients (N = 51)
Demographic features	
Sex (female)	29 (56.9%)
Age (years)	64 ± 12
Body mass index (kg/m^2^)^a^	27.3 (IQR 24.3–31.3)
Acromegaly characteristics	
Duration of active disease (years)^a^	6.5 (IQR 3.0–10.6)
Duration of remission (years)^b^	18.3 (IQR 7.2–25.4)
Treatment strategy	
Surgery	22 (43.1%)
PharmaT	5 (9.8%)
Surgery + PharmaT	14 (27.5%)
Surgery + RT	6 (11.8%)
Surgery + RT + PharmaT	3 (5.9%)
RT + PharmaT	1 (2.0%)
Current pharmacological treatment	
None	32 (62.7%)
SMS analogues	11 (21.6%)
PegV	3 (5.9%)
SMS analogues + PegV	3 (5.9%)
SMS analogues + DA	2 (3.9%)
GH (ug/L)	
Pre-treatment^c^	27.4 (IQR 10.4–43.8)
Current^d^	1.4 (IQR 0.6–6.0)
IGF-1 (nmol/L)	
Pre-treatment^d^	60.0 (IQR 49.0–87.4)
SDS^d^	6.1 (IQR 5.0–7.8)
Current^d^	16.8 ± 4.9
SDS^d^	0.6 ± 1.0

Values are reported as N (%), mean ± SD, or median (interquartile range, IQR). Notably, IGF-1 levels were temporarily elevated in two patients; in one patient IGF-1 SDS was 2.5 due to transient recombinant human GH (rhGH) over-replacement, whereas IGF-1 SDS was 2.2 in another patient in the presence of normal glucose-suppressed GH levels

*BMI* body mass index, *RT* radiotherapy, *SMS* somatostatin, *GH* growth hormone, *IGF-1* insulin-like growth factor-1, *SDS* standardized deviation score, *IQR* interquartile range, *N* number of patients, *WHR* wait-to-hip ratio, *SD* standard deviation

^a^Data available in 47 patients

^b^Data available in 48 patients

^c^Data available in 40 patients

^d^Data available in 37 patients

### Standardized interview and questionnaires

#### Self-reported joint symptoms during standardized interview

As shown in Table 2, knee pain was the most reported joint symptom [uni- or bilaterally in 26 patients (53.1%)], followed by hip pain [20 patients (40.8%)]. Shoulder pain was reported by nineteen patients (38.0%), whereas shoulder stiffness was reported by six patients (12.2%). Pain or stiffness in the feet was reported by twelve patients (24.5%), of whom eleven patients (21.6%) reported pain (3 unilateral, 8 bilateral).

**Table 2:** Reported joint complaints of the upper and lower limbs

Joint location	Unilateral	Bilateral	Uni- or bilateral
*Shoulder*			
Pain	10 (20.4%)	9 (18.4%)	19 (38.8%)
Stiffness	2 (4.1%)	4 (8.2%)	6 (12.2%)
Pain or stiffness			19 (38.8%)
*Hand*			
Pain	3 (6.1%)	16 (32.7%)	19 (38.8%)
Stiffness	5 (10.2%)	8 (16.3%)	13 (25.5%)
Pain or stiffness			24 (49.0%)
*Hip*			
Pain	8 (16.3%)	12 (24.5%)	20 (40.8%)
Stiffness	1 (2.0%)	2 (4.1%)	3 (6.1%)
Pain or stiffness			21 (42.9%)
*Knee*			
Pain	13 (26.5%)	13 (26.5%)	26 (53.1%)
Stiffness	4 (8.2%)	11 (22.4%)	15 (30.6%)
Pain or stiffness			29 (59.2%)
*Ankle*			
Pain	5 (10.2%)	4 (8.2%)	9 (18.4%)
Stiffness	1 (2.0%)	0 (0.0%)	1 (2.0%)
Pain or stiffness			10 (20.4%)
*Foot*			
Pain	3 (6.1%)	8 (16.3%)	11 (22.4%)
Stiffness	0 (0.0%)	3 (6.1%)	3 (6.1%)
Pain or stiffness			12 (24.5%)

Joint complaints, as reported during the standardized interview. Values are reported as N (%). Data were reported for 49 patients

#### Joint-specific questionnaires on self-reported joint symptoms

Median total AUSCAN scores were 6 (IQR 1–20), and total WOMAC scores were 37 (IQR 8–100), indicating mild to moderate disability of the hands and lower limbs, respectively (Supplementary Table 1). Median total DASH score as measure of upper limb disability was 9 (IQR 3–27), with individual scores ranging from 0 (no disability) to 54 (moderate disability) (Supplementary Table 1).

#### Impact of joint complaints on health-related quality of life

Median PCS, and MCS were 45 (IQR 39–49), and 56 (IQR 51–59), respectively (Supplementary Table 1). With respect to the relationship between joint disability and HR-QoL, upper limb disability (i.e. DASH scores) were negatively correlated with PCS, but not with MCS (PCS: r = − 0.638, *P* < 0.0001; MCS: r = − 0.214, *P* = 0.168). Similarly, hand disability (AUSCAN scores), and lower limb disability (WOMAC scores) were solely negatively associated with PCS (AUSCAN: r = − 0.562, *P* < 0.0001; WOMAC: r = − 0.789, *P* < 0.0001), but not with MCS (AUSCAN: r = − 0.088, *P* = 0.571; WOMAC: r = − 0.015, *P* = 0.924).

### Radiographic OA and associated risk factors

#### Radiographic OA of knee, hip and hand joints

**Fig. 3:**
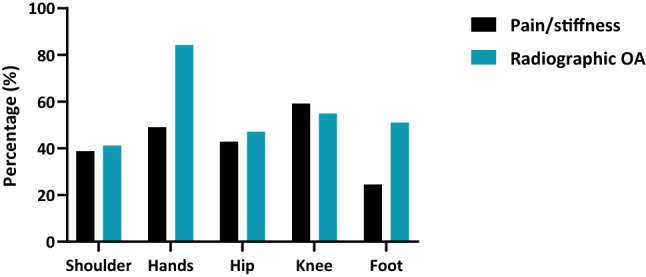
Prevalence of self-reported pain and stiffness, and radiographic OA in all assessed joints. The presence of self-reported joint symptoms (viz*.* pain and/or stiffness) and radiographic OA was assessed. Values are reported as N (%). Data for self-reported joint symptoms were available for 49 patients, whereas radiographic OA data was available for 51 patients. *OA* osteoarthritis

As published in our previous reports [2, 3, 7, 13, 14, 27], radiographic OA of the hands was observed most frequently, namely in 43 patients (84.3%), followed by radiographic knee (28 patients; 54.9%), and hip OA (24 patients; 47.1%), as summarized in Table 3. Three, and eight patients, respectively, had undergone knee (2 unilateral; 1 bilateral), and hip replacement surgery (6 unilateral; 2 bilateral) previously. In Fig. 3, the prevalence of clinical and radiographic OA of the assessed joints is shown.

**Table 3:** Radiographic OA of previously, and newly investigated joint sites in acromegaly

	None	Unilateral	Bilateral	Uni- or bilateral
*Shoulder*				
Glenohumeral	30 (58.8%)	7 (13.7%)	14 (27.5%)	21 (41.2%)
*Hand*				
CMC1	29 (56.9%)	11 (21.6%)	11 (21.6%)	22 (43.1%)
MCPs	25 (49.0%)	7 (13.7%)	19 (37.3%)	26 (51.0%)
PIPs^a^	11 (21.6%)	11 (21.6%)	29 (56.9%)	40 (78.4%)
DIPs^b^	14 (27.5%)	8 (15.7%)	29 (56.9%)	37 (72.5%)
*Hip*	27 (52.9%)	12 (23.5%)	12 (23.5%)	24 (47.1%)
*Knee*	23 (45.1%)	14 (27.5%)	14 (27.5%)	28 (54.9%)
*Feet*				
MTP1	25 (49.0%)	11 (21.6%)	15 (29.4%)	26 (51.0%)
IP1	44 (86.3%)	3 (5.9%)	4 (7.8%)	7 (13.7%)
MTP2-5^c^	49 (96.1%)	1 (2.0%)	1 (2.0%)	2 (3.9%)

The presence of radiographic OA was assessed in established and novel joints based on (modified) KL scoring systems. Values are reported as N (%). Data available for 51 patients

*CMC* carpometacarpal, *DIP* distal interphalangeal, *IP* interphalangeal, *MCP* metacarpophalangeal, *MTP* metatarsophalangeal, *PIP* proximal interphalangeal

^a^For the assessment of PIPs, the IP1 and PIP2-5 joint were combined

^b^The assessment of DIP2-5 was included for DIPs

^c^Of the 2 patients with radiographic OA, one patient had bilateral radiographic OA of the MTP2 joint, and one patient had unilateral radiographic OA of MTP3 joint

#### Radiographic glenohumeral OA

Radiographic glenohumeral OA was observed in 21 patients [41.2%; unilateral: 7 patients (13.7%), bilateral: 14 patients (27.5%)] (Table 3). Median total glenohumeral KL scores were 1 (IQR 0–4). One patient had previously undergone unilateral shoulder replacement surgery. Albeit insignificantly, glenohumeral KL scores appeared higher in patients with self-reported shoulder pain and/or stiffness [median 3 (IQR 0–7) *vs* median 0 (IQR 0–4), *P* = 0.114], and correlated positively with DASH scores reflecting upper limb function (r = 0.32, *P* = 0.03).

Out of established risk factors in primary OA [56–59], solely age [OR 1.23 (1.06–1.43), *P* = 0.007] was significantly associated with radiographic glenohumeral OA. Following correction for age, higher pre-treatment IGF-1 levels were associated with an increased risk of radiographic glenohumeral OA [OR 1.06 (1.01–1.12), *P* = 0.021] (Supplementary Table 2). Moreover, patients currently receiving pharmacological treatment (N = 28), compared to patients cured by surgery and/or radiotherapy (N = 23), had an independently increased risk for radiographic glenohumeral OA [OR 5.82 (1.20–28.34), *P* = 0.029].

#### Radiographic forefoot OA

Radiographic OA of the MTP1 joint was observed in 26 patients (51.0%), of whom 11 patients (21.6%) had unilateral OA, and 15 patients (29.4%) showed bilateral OA (Table 3). Concomitant symptomatic and radiographic OA of the MTP1 joint was observed in ten patients (19.6%). Median total KL score for all assessed forefoot joints was 3 (IQR 2–5).

General, or acromegaly-related risk factors for radiographic MTP1 OA were not detected (Supplementary Table 2) [60, 61]. Because of the low prevalence of radiographic OA in MTP2-5, and IP1 joints, no risk factor analyses were not performed for these joints.

### Generalized versus localized radiographic OA

Forty-six patients (90.2%) had radiographic OA at ≥ 1 joint site, indicating that only a minority of controlled acromegaly patients had no evidence of peripheral radiographic OA. Of the 46 patients with radiographic OA, solely 2 patients (4.3%) had localized radiographic OA at only 1 joint site (both unilateral MTP1 OA). The remaining 44 patients (95.7%) suffered from generalized OA (i.e. ≥ 2 joints, including multiple joints in hands).

### Radiographic OA severity at different joint locations

Severity of radiographic OA at upper limb joint locations, viz*.* glenohumeral and hands, were significantly associated (r = 0.60, *P* < 0.0001). Strikingly, severity of radiographic OA at lower limb joint locations (hip, knee, and forefoot) were not associated. Moreover, severity of radiographic hand OA was associated with the severity of forefoot OA (r = 0.39, *P* = 0.004), and severity of knee OA was associated with severity of glenohumeral OA (r = 0.42, *P* = 0.002).

### Peripheral radiographic OA severity

In the present population, total peripheral radiographic OA scores were 39 ± 27 (range 0–111), indicating a varying degree of severity of radiographic OA of the peripheral joints. Active disease duration (β = 1.09 ± 0.44, *P* = 0.018), and pre-treatment IGF-1 levels (β = 0.27 ± 0.13, *P* = 0.043) were significant predictors for higher severity of peripheral radiographic OA following correction for age and sex (Supplementary Table 3). Notably, severity of peripheral radiographic OA did not differ between patients with hypopituitarism (38 ± 23) or without hypopituitarism (40 ± 30, *P* = 0.862).

### Associations between HR-QoL and severity of radiographic OA

Severity of peripheral radiographic OA was negatively associated with physical HR-QoL (PCS: r = − 0.32, *P* = 0.032), but not with mental HR-QoL (MCS: r = − 0.18, *P* = 0.255). Physical HR-QoL was significantly negatively associated with the severity of radiographic hand OA (hand: r = − 0.31, *P* = 0.039). Albeit insignificantly, the severity of radiographic glenohumeral, hip and knee OA appeared to be associated with lower physical HR-QoL (glenohumeral: r = − 0.28, *P* = 0.062; hip: r = − 0.29, *P* = 0.057; knee: r = − 0.28, *P* = 0.07). However, following correction for age, sex, and BMI [62], the combined radiographic OA severity of all peripheral joints was not an independent risk factor for physical HR-QoL.

## Discussion

In the present study in controlled acromegaly, joint complaints were reported frequently in previously-assessed peripheral joints (viz*.* hands, hips, knees). Additionally, shoulder complaints were common, whereas feet complaints were reported less frequently. Over 90% of patients showed radiographic signs of OA at any peripheral joint site, which occurred in a generalized manner in virtually all patients. Radiographic MTP1 OA was as prevalent as radiographic knee OA, whereas radiographic glenohumeral OA was similarly prevalent as hip OA. Higher pre-treatment IGF-1 levels, and current pharmacological treatment were identified as risk factors for radiographic glenohumeral OA, whereas no risk factors for MTP1 joint OA could be identified.

The high prevalence of acromegalic arthropathy—investigating hips, knees, hands, and spine joints—has been described extensively [2, 3, 6, 7, 13–15]. When investigating these previously-assessed joints in the present population, the mismatch between clinical symptoms and the presence of radiographic abnormalities was noted, which differed from the previously reported prevalence in some joints (e.g. hips) [63]. This difference might be due to the selected population of clearly well-controlled patients with years-long normal circulating GH, and IGF-1 levels at the time of the study visit.

Studies systematically investigating acromegalic arthropathy of other peripheral joints were lacking. Because of the (potential) invalidating nature of shoulder and forefoot OA, the present study investigated whether these joints were affected in acromegaly [16, 18, 25]. Moreover, both in patients with acromegaly, and primary OA, (radiographic) OA can be limited to one specific joint (viz*.* mono-articular), or generalized (viz*.* polyarticular) [61], which could only be investigated by assessing several peripheral joints simultaneously. Virtually all patients in our controlled acromegaly cohort had generalized radiographic OA, with solely two patients having localized MTP1 OA, underlining the differences between systemic causes of OA (i.e. GH excess), and biomechanical factors (e.g. strenuous use of one joint).

When assessing the shoulder joint in the present population of patients with controlled acromegaly, prevalence of self-reported complaints varied from 12.2% (stiffness) to 38.8% (pain), being consistent with previous reports, although previous studies were mainly performed in active disease [20, 21]. Moreover, higher disability scores (using the validated DASH questionnaire as a measure for upper limb disability) were reported by our patients with acromegaly in remission compared to the general population [64], although scores were lower than reported scores in patients with carpal tunnel syndrome [65], or rheumatoid arthritis [66]. Moreover, disability of the upper limb was negatively associated with HR-QoL in the present study, highlighting the invalidating nature of shoulder complaints.

For the first time, the characteristic radiographic features of acromegalic arthropathy were described in the glenohumeral joint [9–12, 67], with radiographic glenohumeral OA being present in 41.2% of controlled patients. This radiographic OA prevalence is higher than the prevalence of radiographic OA at other large joint sites [2, 3, 6, 7, 13–15], and higher than in the general population, although the latter varied greatly depending on the cohort and scoring methods across different studies [56, 68–70].

Although age, female sex, and BMI are established risk factors for radiographic glenohumeral OA in the general population [56–59], solely age was identified as a risk factor in the present population. Furthermore, higher pre-treatment IGF-1 levels, and current use of pharmacological treatment to achieve remission were associated with an increased risk of glenohumeral OA. These findings are in accordance with our previous studies, showing a significant relationship between acromegaly-specific risk factors, and the presence and progression of radiographic OA of the spine, hip, knee and hand joints [13, 71, 72], indicating the partial systemic nature of glenohumeral OA.

With respect to forefoot OA, we observed a comparable prevalence of self-reported pain or stiffness of the feet in controlled acromegaly, and the general population. To date, foot complaints have never been systematically assessed in patients with acromegaly, except for a few ultrasound studies focusing on tendinopathy [22, 73]. Complaints of the feet increase the demand of care, since almost 10% of all musculoskeletal consultations with general practitioners comprise foot (or ankle) pain [74], which have detrimental effects on daily functioning and QoL [24, 25].

Radiographic OA of the MTP1 joint was observed in over half of the controlled acromegaly patients, thereby being much more prevalent than radiographic OA at other peripheral joint sites in patients with acromegaly [2, 3, 6, 7, 13–15]. By contrast, radiographic OA of the MTP2-5, and IP1 joint occurred infrequently. In the general population, radiographic OA of the MTP1 joint ranged from 5.0% to 42.0%, and 3.0% to 4.9% for the MTP2-5 joints, depending on age, sex, and country of origin [60]. Prevalence of radiographic OA of the MTP1 joint in Dutch individuals ranged from 31.4% for ages 55–59 years to 44.4% for ages > 80 years [70], which is lower than the prevalence observed when biochemical disease control is reached in patients with acromegaly. In the present study, however, the exact clinical significance of the presence of foot complaints and radiographic OA cannot be determined in the absence of validated questionnaires. Nonetheless, the high prevalence of radiographic OA of the MTP1 joint combined with the reported clinical symptoms, as well as the association between foot deformities (e.g. hallux valgus) and radiographic MTP1 OA [60], the MTP1 joint is an important joint to evaluate in acromegaly patients.

Established risk factors for radiographic MTP1 OA in the general population are higher age, female sex and increased BMI [60, 61, 75], albeit these factors could not be identified in patients with acromegaly. Moreover, measures of disease activity/severity were not detected as risk factors for MTP1 OA. Additional factors related to education, and occupation, e.g. lower educational attainment, and (a history of) physically demanding occupation (e.g. frequent stair climbing, professional dance) appear to contribute more to the risk of MTP1 clinical or radiographic OA in the general population, and primary OA [54, 75–77], and we therefore assume that the combination of biomechanical factors, including acromegaly-related physical changes (e.g. feet enlargement, joint misalignment), contribute most to the development of MTP1 OA in acromegaly. We advise to structurally assess the foot joints in acromegaly patients, since more stringent disease control is not likely to improve the foot complaints, whereas a multitude of other interventions are available.

**Fig. 4:**
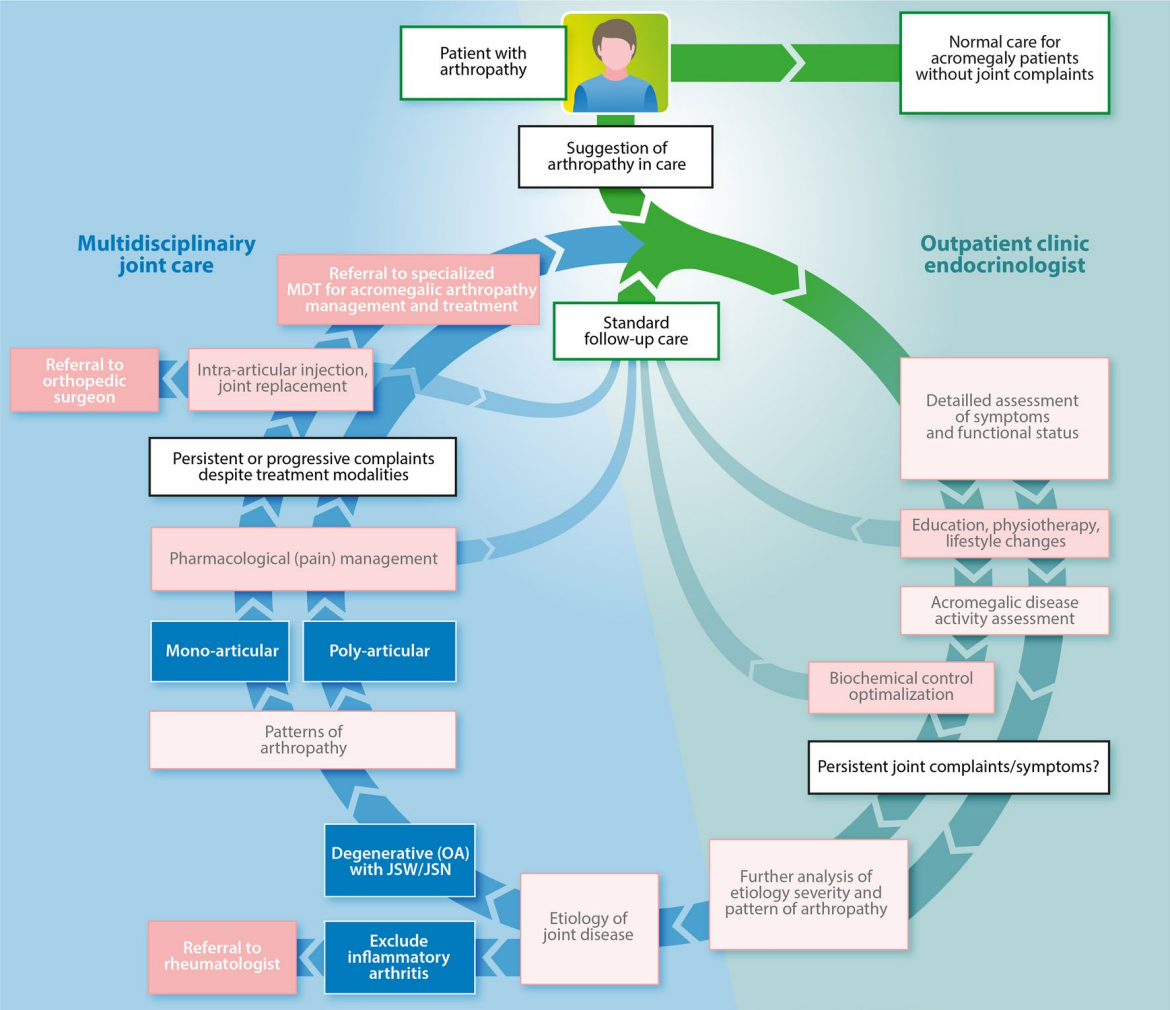
Flowchart of diagnostic and treatment algorithm for joint-related disability in patients with acromegaly. Based on clinical expertise and performed clinical studies, we propose an algorithm for the diagnosis and subsequent treatment and management of patients with acromegaly with joint-related disability in a Pituitary Center of Excellence (PTCOE) [79]. The cornerstone of treatment remains adequate biochemical disease control and the next steps in the care path depend on the etiology, severity and extensiveness of acromegalic arthropathy. *JSN* joint space narrowing, *JSW* joint space widening, *MDT* multidisciplinary team, *OA* osteoarthritis

To date, acromegalic arthropathy cannot be prevented, and its optimal management remains to be elucidated [15, 78]. We propose a diagnostic and therapeutic algorithm regarding the management of joint complications in acromegaly in Fig. 4, based on our extensive clinical expertise and research. All patients with acromegaly should be diagnosed, treated, and followed at a pituitary center of excellence (PTCOE) [79], of which the exact implementation is dependent on the (inter)national organization of health care. For acromegaly care, an exemplary PTCOE would harbor a multidisciplinary team (MDT) of an endocrinologist, neurosurgeon, rheumatologist, radiologist, orthopedic surgeon, physical therapist, and dietician. Stringent GH/IGF-1 hypersecretion control remains the cornerstone of acromegaly management, since smoldering disease might cause cartilage loss, and arthropathy progression [13, 15, 72]. Assessment of joint complaints should be performed regularly, preferably using validated questionnaires. In the case of joint symptoms, referral to MDT members might be necessary for adequate diagnostics, of which the exact route depends on the etiology of (osteo)arthritis and extensiveness of affected joints. In this respect, there should be a focus on etiologies for which effective treatment strategies are available, such as neuropathic pain [26], and inflammatory rheumatic disease [80]. Unfortunately, treatment strategies for acromegalic arthropathy are mostly symptomatic, and similar to treatment options for primary OA, including lifestyle advice, analgesics, physical therapy, intra-articular corticosteroid injections, or joint replacement therapy [81, 82]. Notably, none of these treatment strategies have been formally studied in acromegalic arthropathy to date. In case of persistent joint-related disability despite adequate biochemical disease control, and first-line treatment, we advise discussion in MDT meetings to evaluate an individualized, personalized strategy.

Several limitations of this study need to be addressed. First, although the patient population potentially was relatively small, this study describes a unique cohort of acromegaly patients in remission, and is the first study describing both clinical and radiographic OA in the shoulder and feet. Modified KL atlases based on the existing KL atlases of the knee and hand were used for the glenohumeral and MTP joints, similar to previous studies [49–55]. No (modified) KL atlas was available for the acromioclavicular and sternoclavicular joints, and, therefore, as mentioned prior, solely the glenohumeral joint of the shoulder was assessed. Additionally, the unavailability of a widely-used KL atlas, as well as the unavailability of an Osteoarthritis Research Society International (OARSI) atlas of the shoulder and foot (a scoring method in which the radiographic joint abnormalities, e.g. JSN and OP, are individually assessed), hampered the comparison of patients with acromegaly to healthy controls, since assessed radiographic glenohumeral and forefoot OA (age-matched) healthy controls were unavailable. Moreover, the etiology and accompanying characteristics of the subsets of patients with JSN or JSW cannot be assessed at this time. Finally, validated questionnaires for feet complaints are unfortunately lacking, and therefore not included in this study.

In conclusion, for the first time, a high prevalence of both self-reported joint complaints and radiographic OA of newly assessed peripheral joint sites in patients with controlled acromegaly is reported. Whereas the direct effects of transient GH/IGF-1 excess are assumed to cause acromegalic arthropathy at most joint sites, resulting in a generalized (radiographic) OA pattern, primarily biomechanical factors play a role in the development of MTP1 joint OA. Available treatment options of acromegalic arthropathy remain symptomatic, and should be the focus of future studies, representing an important unmet need in the current care of acromegaly patients.

## Supplementary Information

Below is the link to the electronic supplementary material.

Supplementary file1 (DOCX 17 KB)

Supplementary file2 (DOCX 17 KB)

Supplementary file3 (DOCX 17 KB)
