# Supplementary material for: Clinical and radiographic assessment of peripheral joints in controlled acromegaly
Source: Pituitary. 2022 Jun 20;25(4):622–35. doi: 10.1007/s11102-022-01233-z (PMC9345810; doi:10.1007/s11102-022-01233-z)
Supplement: Supplementary file 1 — Supplementary file1 (DOCX 17 KB) [file 11102_2022_1233_MOESM1_ESM.docx]

**Supplementary Table 1**

| Parameter |  | Range | All patients (N=51) |
| --- | --- | --- | --- |
| *HR-QoL** | Physical Component Score | 0 – 100 | 45 (IQR 39 - 49) |
|  | Mental Component Score | 0 – 100 | 56 (IQR 51 - 59) |
| *DASH*** | Total score | 0 – 100 | 9 (IQR 3-27) |
| *AUSCAN**** | Total | 0 – 60 | 6 (IQR 1 – 20) |
|  | Pain | 0 – 20 | 2 (IQR 0 – 6) |
|  | Stiffness | 0 – 4 | 0 (IQR 0 – 1) |
|  | Function | 0 – 36 | 3 (IQR 0 – 12) |
| *WOMAC* | Total**** | 0 – 300 | 37 (IQR 8 – 100) |
|  | Pain*** | 0 – 100 | 10 (IQR 3 – 32) |
|  | Stiffness*** | 0 – 100 | 15 (IQR 3 – 38) |
|  | Function**** | 0 – 100 | 8 (IQR 3 – 28) |

**Supplementary Table 1 – Reported HR-QoL and disability of the upper and lower limbs**

Disability of the upper limb, as assessed using the DASH and AUSCAN questionnaires, lower limb disability as assessed using the WOMAC, and health-related quality of life (HR-QoL), as assessed by the SF-36 questionnaire are shown. Values are reported as N (%), or median (IQR). * Data available in 47 patients, ** Data available in 46 patients, *** Data available in 48 patients, **** Data available for 41 patients.

AUSCAN, Australian/Canadian Osteoarthritis Index; DASH, Disabilities of the Arm, Shoulder and Hand; SF-36, Short Form-36; WOMAC, Western Ontario and McMaster Universities Osteoarthritis Index
