# Supplementary material for: Clinical and radiographic assessment of peripheral joints in controlled acromegaly
Source: Pituitary. 2022 Jun 20;25(4):622–35. doi: 10.1007/s11102-022-01233-z (PMC9345810; doi:10.1007/s11102-022-01233-z)
Supplement: Supplementary file 2 — Supplementary file2 (DOCX 17 KB) [file 11102_2022_1233_MOESM2_ESM.docx]

**Supplementary Table 2**

| Parameter | Radiographic glenohumeral OA | | Radiographic MTP1 OA | | |
| --- | --- | --- | --- | --- | --- |
|  | **OR (95% CI)** | **P-value** | | **OR (95% CI)** | **P-value** |
| Active disease duration (years)* | 1.06 (0.95 – 1.18) | 0.282 | | 1.01 (0.93 – 1.10) | 0.824 |
| Treatment modality ** | 5.01 (1.03 – 24.54) | 0.047 | | 1.26 0.42 – 1.38 | 0.683 |
| Pre-treatment IGF-1 level (nmol/L)*** | 1.06 (1.01 – 1.12) | 0.021 | | 1.00 (0.97 – 1.02) | 0.688 |
| Current IGF-1 level (nmol/L)*** | 0.95 (0.82 – 1.10) | 0.463 | | 0.94 (0.83 – 1.06) | 0.303 |

**Supplementary Table 2 – Risk factors for radiographic glenohumeral and MTP1 OA**

For both glenohumeral and MTP1 joint radiographic OA, logistic regression analyses were performed to assess specific risk factors. Values are reported as Odds ratios (OR) with 95% confidence intervals (CI). * Available for 47 patients, ** Treatment modality is defined as currently being in remission due to pharmaT, ***Available for 37 patients.
