# Supplementary material for: Clinical and radiographic assessment of peripheral joints in controlled acromegaly
Source: Pituitary. 2022 Jun 20;25(4):622–35. doi: 10.1007/s11102-022-01233-z (PMC9345810; doi:10.1007/s11102-022-01233-z)
Supplement: Supplementary file 3 — Supplementary file3 (DOCX 17 KB) [file 11102_2022_1233_MOESM3_ESM.docx]

**Supplementary Table 3**

| Parameter | Severity of radiographic peripheral OA | |  |
| --- | --- | --- | --- |
|  | **β±SEM** | **P-value** | |
| Active disease duration (years)* | 1.09 ± 0.44 | 0.018 | |
| Treatment modality ** | 8.16 ± 5.97 | 0.177 | |
| Pre-treatment IGF-1 level (nmol/L)*** | 0.27 ± 0.13 | 0.043 | |
| Current IGF-1 level (nmol/L)*** | -1.23 ± 0.63 | 0.059 | |

**Supplementary Table 3 – Risk factors for severity of peripheral radiographic OA**

Linear regression analyses were performed to assess specific risk factors for severity of peripheral radiographic OA, as indicated by mean total Kellgren and Lawrence scores for all assessed joints ranging from 0-192, correcting for age and sex. Values are reported as β and standard errors of the mean (SEM). * Available for 47 patients, ** Treatment modality is defined as currently being in remission due to pharmacological treatment, ***Available for 37 patients.
